# Klinische Ergebnisse der Ellenbogentotalendoprothetik nach Therapieversagen bei Monteggia-like-Verletzungen

**DOI:** 10.1007/s00113-023-01335-8

**Published:** 2023-06-04

**Authors:** Jan Hockmann, Nadine Ott, Tim Leschinger, Lars Peter Müller, Michael Hackl

**Affiliations:** Universität zu Köln, Medizinische Fakultät und Uniklinik Köln, Klinik für Orthopädie, Unfallchirurgie und Plastische Chirurgie, Kerpener Straße 62, 50937 Köln, Deutschland

**Keywords:** Ellenbogen, Monteggia, Frakturfolgezustände, Endoprothese, Komplikationen, Elbow, Monteggia, Fracture, Arthroplasty, Complications

## Abstract

**Hintergrund:**

Monteggia-like-Frakturen (MLF) sind komplexe Verletzungen mit hohen Komplikationsraten und häufig unbefriedigenden funktionellen Ergebnissen. Bei posttraumatischer Gelenkdestruktion nach MLF stellt die Implantation einer Ellenbogentotalendoprothese (EB-TEP) oftmals die einzige Option zum Erhalt der Funktionalität des Ellenbogens dar. Diese Fallserie beleuchtet die klinischen Ergebnisse der EB-TEP nach gescheiterter Behandlung einer MLF.

**Methoden:**

Alle Patienten mit EB-TEP-Implantation (2017–2022) nach Therapieversagen bei MLF wurden retrospektiv eingeschlossen. Die Komplikationen sowie Revisionen vor und nach EB-TEP und das funktionelle Ergebnis, bemessen mittels Broberg and Morrey-Score, wurden evaluiert.

**Ergebnisse:**

In diese Studie wurden 9 Patienten mit einem Alter zum Zeitpunkt der EB-TEP-Implantation von 68 (±7; 54 bis 79) Jahren inkludiert. Der Nachbetrachtungszeitraum betrug 12 (±9; 2 bis 27) Monate. Die wesentlichen Ursachen, die zur Gelenkdestruktion führten, waren die chronische Osteomyelitis (44,4 %), die knöcherne Instabilität durch einen Processus-coronoideus-Defekt (33,3 %) bzw. einen kombinierten Processus-coronoideus- und Radiuskopfdefekt (22,2 %) sowie die Pseudarthrose der proximalen Ulna mit Radiuskopfnekrose (11,1 %). Die Anzahl an Revisionsoperationen von der Primärversorgung bis zur EB-TEP lag bei 2,7 (±1,8; 0 bis 6) Eingriffen. Die Revisionsrate nach EB-TEP betrug 44 %. Der Broberg/Morrey Score lag zum Zeitpunkt der letzten Nachuntersuchung bei 83 (±10; 71 bis 97) Punkten.

**Zusammenfassung:**

Die chronische Osteomyelitis und der Defekt des Processus coronoideus sind die führenden Ursachen für eine Gelenkdestruktion nach MLF, die zur EB-TEP führen. Wenngleich die klinischen Ergebnisse insgesamt zufriedenstellend sind, so muss angesichts der hohen Revisionsrate die Indikationsstellung streng selektiert werden.

## Abstract (en)

**Background:**

Monteggia-like fractures (MLF) are complex injuries with high complication rates and frequently unsatisfying clinical results. Total elbow arthroplasty (TEA) represents the only option to salvage the functional requirements in some patients with pronounced posttraumatic arthropathy. This case series provides clinical results of TEA following failed treatment of MLF.

**Methods:**

All patients who underwent TEA from 2017 to 2022 for failed treatment of MLF were retrospectively included in this study. Complications and revisions before and after TEA and functional results, measured by the Broberg/Morrey score, were evaluated.

**Results:**

In this study 9 patients with a mean age of 68 (±7; 54–79) years were included. The mean follow-up was 12 (±9; 2–27) months. The main reasons leading to posttraumatic arthropathy were chronic infections (44.4%), bony instability due to coronoid deficiency (33.3%) or a combined coronoid and radial head deficiency (22.2%) and non-union of the proximal ulna with radial head necrosis (11.1%). The mean number of surgical revisions between primary fixation and TEA was 2.7 (±1.8; 0–6). The revision rate following TEA was 44%. The mean Broberg/Morrey score at the time of the latest follow-up was 83 (±10; 71–97) points.

**Conclusion:**

Chronic infection and coronoid deficiency are the main reasons for posttraumatic arthropathy after MLF leading to TEA. While the overall clinical results are satisfying, the indications should be reserved for selected cases due to high revision rates.

## Einleitung

Die Monteggia-like-Verletzung, auch bekannt als Monteggia-Äquivalent Verletzung, ist eine Kombinationsverletzung des Ellenbogengelenkes aus einer proximalen Ulnafraktur mit Luxation oder Luxationsfraktur des Radiuskopfes aus dem proximalen, radioulnaren Gelenk [1, 20]. Insbesondere aufgrund der geringen Prävalenz von nur 0,7–5 % aller Ellenbogenverletzungen ist das dezidierte Verständnis der anatomischen Strukturen Grundvoraussetzung für ein erfolgreiches Therapiemanagement [8, 17, 22]. Der zugrunde liegende Verletzungsmechanismus ist entweder ein Hochenergietrauma mit direkter Krafteinwirkung auf den Ellenbogen oder auch ein Sturz auf den ausgestreckten Arm; Letzterer v. a. beim geriatrischen Patienten [12, 16]. Das Niedrigenergietrauma beim geriatrischen Patienten gewinnt klinisch zunehmend an Bedeutung und stellt den Operateur vor eine besonders große Herausforderung [3].

**Figure 1:**
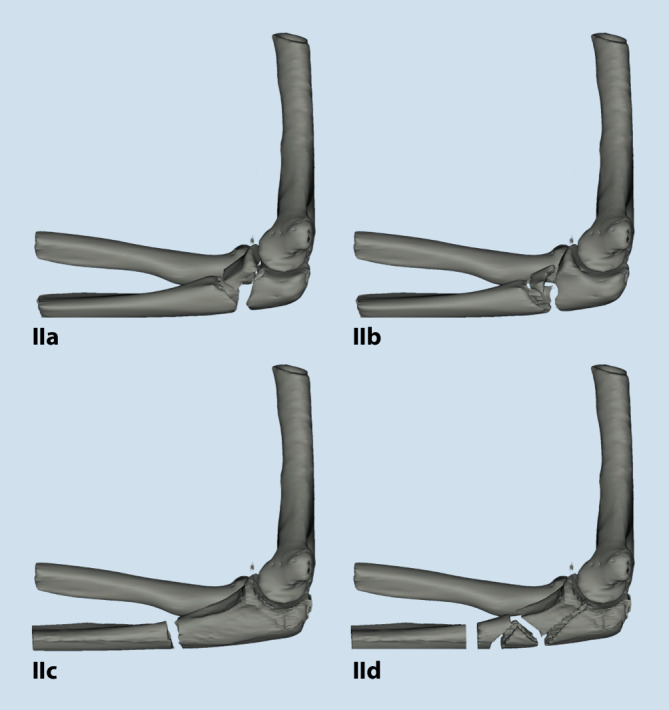

Die Monteggia und Monteggia-like-Frakturen werden nach Bado klassifiziert. Für die beim erwachsenen Patienten typische Bado-Typ-II-Verletzung mit dorsaler Luxation bzw. Luxationsfraktur des Radiuskopfes existiert eine Subklassifikation nach Jupiter, die zwar im Alltag gebräuchlich ist, jedoch keine Handlungsempfehlung geben kann ([1, 15, 17]; Abb. 1). Aufgrund der Komplexität dieser Verletzungen ist daher eine Computertomographie mit 3D-Rekonstruktion zur präoperativen Planung sinnvoll.

Das Ziel der operativen Versorgung ist die anatomische Reposition mit Stabilisierung des Ellenbogengelenkes, um möglichst eine frühfunktionelle Rehabilitation zu ermöglichen [5]. Da es sich um eine Luxationsfraktur handelt, gilt der kongruenten Stellung des Radiuskopfes zum Capitulum humeri sowie dem proximalen Radioulnargelenk ein besonderes Augenmerk. Entscheidend für die kongruente Gelenkstellung ist die anatomische Rekonstruktion der proximalen Ulna sowie des Processus coronoideus.

Die direkte Implantation einer Ellenbogentotalendoprothese bleibt in der Akutsituation Ausnahmefällen vorbehalten [13, 14]. Bei posttraumatischer Gelenkdestruktion stellt der prothetische Gelenkersatz jedoch oftmals die einzige Option dar, um die Funktionalität der Extremität zu erhalten. Das klinische Outcome der Ellenbogentotalendoprothese nach Monteggia-like-Fraktur ist in der Literatur nur spärlich berichtet [25].

Daher war das Ziel dieser retrospektiven Studie, die klinischen Ergebnisse nach Implantation einer Ellenbogentotalendoprothese infolge einer gescheiterten Behandlung nach Monteggia-like-Fraktur zu untersuchen.

## Methoden

Retrospektiv wurden alle Patienten ≥ 18 Jahren erfasst, bei denen im Schwerpunkt Unfall‑, Hand- und Ellenbogenchirurgie der Klinik für Orthopädie, Unfallchirurgie und Plastische Chirurgie der Uniklinik Köln im Zeitraum von 2017 bis 2022 infolge einer Revisionsoperation nach Monteggia-like-Verletzung eine Ellenbogentotalendoprothese implantiert wurde. Retrospektive wurden Arztbriefe, Befundberichte aus der spezifischen Sprechstunde, Operationsberichte, mikrobiologische Befunde sowie die vorliegende Bildgebung evaluiert. Anhand der Befundberichte wurde der Score nach Broberg und Morrey berechnet [4].

Die statistische Auswertung wurde mittels IBM SPSS Statistics Version 29.0 (IBM Corp, Armonk, NY) vorgenommen. Die Daten werden dargestellt als Mittelwert mit Standardabweichung und Spannweite (Standardabweichung; Minimum bis Maximum).

## Ergebnisse

### Patientenkollektiv

Im Zeitraum von 2017–2022 konnten insgesamt 9 Patienten eingeschlossen werden. Hiervon waren 6 weiblich und 3 männlich. Das durchschnittliche Alter lag bei 68 (±7; 54 bis 79) Jahren. Die Frakturen ließen sich nach Bado und Jupiter klassifizieren als Typ 2B- (4), Typ 2D- (4) und Typ 2C-Verletzung (1). Zwei Patienten präsentierten sich initial mit einer erstgradig offenen Fraktur nach Gustilo und Anderson [10]. Es konnte ein durchschnittlicher Nachbeobachtungszeitraum nach Ellenbogentotalendoprothesenimplantation von 12 (±9; 2 bis 27) Monaten erreicht werden.

### Operative Versorgung

Für die Erstversorgung erhielten 2 Patienten einen Fixateur externe. Bei allen Patienten wurde im Rahmen der Definitivversorgung eine Plattenosteosynthese der proximalen Ulna durchgeführt. Eine zusätzliche Osteosynthese des Radiuskopfes erfolgte in 4 Fällen, eine Radiuskopfprothese wurde in einem Fall implantiert. In 2 Fällen wurde der Radiuskopf reseziert.

**Figure 2:**
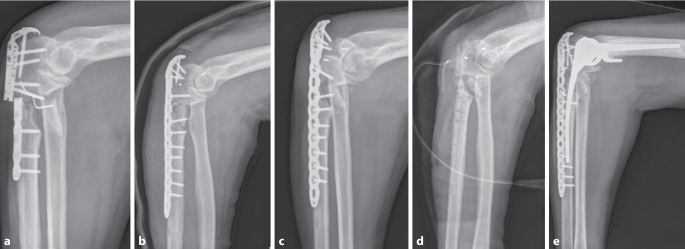

Nach initialer Versorgung lag die durchschnittliche Anzahl an Revisionsoperationen bis zur Implantation der Ellenbogentotalendoprothese bei 2,7 (±1,8; 0–6). Zwischen primärer Osteosynthese und Implantation der Ellenbogentotalendoprothese lagen im Mittel 9 (±6; 1,3 bis 18,6) Monate. Bei allen Patienten wurde eine gekoppelte, zementierte, ulnohumerale Ellenbogentotalendoprothese implantiert; in 6 Fällen mit Langschaft der Ulna (66,7 %), in 3 Fällen mit Standardschaft (33,3 %). In den meisten Fällen (88,9 %) erfolgte eine additive Plattenosteosynthese der Ulna (Abb. 2 und 3). Der verwendete Prothesentyp war in 8 Fällen eine Ellenbogen-TEP ohne Radiuskopfersatz des Modells Latitude der Fa. Tornier/Wright (Memphis, TN, USA). Bei einer Patientin wurde eine Nexel-Prothese der Fa. Zimmer (Zug, Schweiz) verwendet.

**Figure 3:**
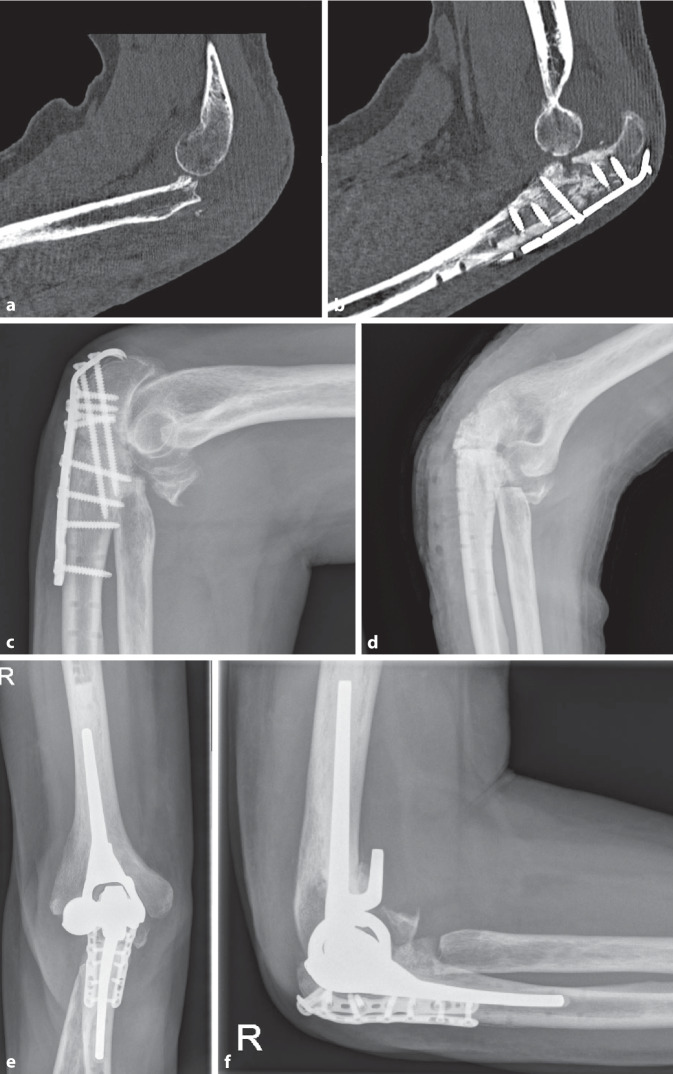

### Komplikationen

Die Komplikationsursachen, die zur Implantation der Ellenbogentotalendoprothese führten, waren 4‑mal (44,4 %) eine chronische Osteomyelitis (2-mal *Cutibacterium acnes*, einmal *Staphylococcus aureus*, einmal *Staphylococcus epidermidis*), 3‑mal (33,3 %) eine knöcherne Instabilität durch einen Defekt des Processus coronoideus ulnae, 2‑mal (22,2 %) eine knöcherne Instabilität durch einen kombinierten Defekt des Processus coronoideus und des Radiuskopfes (davon einmal in Kombination mit einer chronischen Osteomyelitis) und einmal (11,1 %) eine aseptische Pseudarthrose der Ulna mit Radiuskopfnekrose. Nach der primären Osteosynthese gab es in 4 Fällen neurologische Komplikationen. Darunter kam es bei 3 Patienten zur transienten, sensiblen Ulnarisneuropathie und bei einer Patientin zu einer transienten Parese des N. radialis nach Fixateur-Anlage.

Nach Prothesenimplantation kam es in 4 Fällen zu operativen Revisionen (44,4 %). Hierzu zählten die Entlastung eines postoperativen Hämatoms (1), die Resektion von heterotopen proximal radioulnaren Ossifikationen mit Umwendblock (1), die Wundrevision bei subkutanem Infekt (1) und der Wechsel der mobilen Teile bei tiefem Infekt (1).

### Funktionelles Ergebnis

Im Mittel erreichten die Patienten 83 (±10; 71 bis 97) Punkte nach Broberg/Morrey.

## Diskussion

Die vorliegende Studie zeigt, dass die Ellenbogentotalendoprothese bei posttraumatischer Arthropathie nach Monteggia-like-Fraktur zwar insgesamt zufriedenstellende klinische Ergebnisse liefert, jedoch mit einer anhaltend hohen Revisions- und Komplikationsrate vergesellschaftet ist. Entsprechend kritisch sollte die Indikation zum endoprothetischen Gelenkersatz gesehen werden.

Monteggia-like-Verletzungen stellen komplexe Luxationsfrakturen dar, die in der Regel einer dezidierten Rekonstruktion bedürfen, um ein gutes klinisches Ergebnis zu erreichen. Neben der anatomischen Rekonstruktion von Länge, Achse und Rotation der Ulna, die die Grundvoraussetzung für eine kongruente Artikulation des proximal radioulnaren und des radiohumeralen Gelenks ist, gilt es, die Schlüsselfragmente zu identifizieren und zu stabilisieren. Hierzu gehören v. a. Frakturen des Processus coronoideus ulnae, des Radiuskopfes und ulnarseitig knöchern ausgerissene Kollateralbänder am Tuberculum subliminus bzw. an der Crista supinatoria. Trotz anatomisch aufwendiger Rekonstruktionen werden häufig nur unbefriedigende Ergebnisse erreicht [9, 13, 14, 17, 22, 25]. So ist aus dieser Fallserie ersichtlich, dass insbesondere die Vernachlässigung von Frakturen des Processus coronoideus und des Radiuskopfes zur knöchernen Instabilität mit chronischer (Sub‑)Luxationsstellung des Gelenks und rascher Gelenkdegeneration führen kann [5, 16, 18]. Diese Erkenntnis wird gestützt durch die Arbeit von Klug et al. [16], die zuverlässige klinische Ergebnisse nach Monteggia-like-Frakturen publizierten, wenn begleitende Frakturen des Radiuskopfes osteosynthetisch oder – bei nichtrekonstruierbarer Fraktur – mittels Radiuskopfprothese versorgt wurden.

Der prothetische Ersatz des Radiuskopfes im Rahmen von Monteggia-like-Frakturen ist besonders anspruchsvoll, da die übliche Höhenreferenz der dorsalen Kante der Incisura radialis [24] zunächst rekonstruiert werden muss, ehe die Radiuskopfprothesenimplantation erfolgen kann. Insbesondere ein „overlengthening“ der Prothese gilt es, strikt zu vermeiden, da dies zu einer Gelenkinkongruenz und zur Arrosion des Capitulums führt [23, 26]. So konnten Ramazanian et al. in einer biomechanischen Studie demonstrieren, dass bereits ein Overlengthening der Radiuskopfprothese von 2 mm erhöhte Anpressdrücke im radiohumeralen Gelenkspalt und eine verringerte Kontaktfläche des Processus coronoideus verursacht [21].

Dass trotz adäquater Gelenkrekonstruktion nicht selten schlechte klinische Ergebnisse nach Monteggia-like-Frakturen berichtet werden, liegt nicht zuletzt auch an erhöhten Infektionsraten. Begründet sind diese einerseits aus dem verheerenden, oftmals offenen Weichteilschaden und andererseits aus aufwendigen Eingriffen mit langer Operationszeit [6, 7]. Der Anteil an initial offenen Frakturen und chronischen Infektionen in unserem Kollektiv deckt sich dabei mit der verfügbaren Literatur [12, 16].

Weiterhin ist anzumerken, dass durch den demografischen Wandel heutzutage vermehrt ältere, v. a. weibliche Patienten mit reduzierter Knochenqualität derartige Frakturen im Rahmen von Niedrigenergietraumata erleiden [16, 22]. Insbesondere in diesem hauptsächlich weiblichen Kollektiv mit einem durchschnittlichen Alter von 68 Jahren ist eine stabile Rekonstruktion von komplexen Monteggia-like Läsionen häufig erschwert und die Komplikationsrate entsprechend erhöht. Suarez et al. publizierten eine Fallserie von 44 Patienten mit Monteggia-like-Verletzungen, von denen mehr als zwei Drittel eben jenem Patientenkollektiv zuzuordnen waren, und berichteten eine auffallend hohe Komplikationsrate von 48 %. Die häufigsten Komplikationen waren auch hier Infektionen, Pseudarthrosen und Instabilitäten mit häufig begleitendem Materialversagen [22].

Primäre Frakturprothesen am Ellenbogen zeigen allen voran bei nichtrekonstruierbaren distalen Humerusfrakturen des älteren Patienten gute Ergebnisse, wohingegen in der posttraumatischen Situation das klinische Outcome weniger zuverlässig ist [2, 11, 19]. Angesichts der hohen Komplikationsraten nach Osteosynthesen von Monteggia-like-Frakturen sowie nach sekundärer Implantation einer Ellenbogentotalendoprothese stellt sich die Frage, ob die primäre Frakturendoprothetik insbesondere beim geriatrischen Patienten von zunehmender Relevanz sein sollte. Durch die oft komplexe Fraktursituation, die nicht selten bis in die Diaphyse reicht, ist jedoch eine Prothesenimplantation in der akuten Fraktursituation zumeist nur mittels Langschaft und additiver Plattenosteosynthese der Ulna mit Wiederherstellung der Kontinuität der Ulna und v. a. des Streckapparats möglich. Daher bleibt sie bei Monteggia-like-Frakturen nur Ausnahmefällen vorbehalten.

### Limitationen

Diese retrospektive Studie ist durch ihre begrenzte Fallzahl und den kurzen Nachbetrachtungszeitraum limitiert. Das retrospektive Studiendesign und das heterotope Patientenkollektiv lassen nur bedingt Schlüsse zu. Größere, vergleichende, prospektive Studien mit längerem Follow-up sind nötig, um den Stellenwert der Ellenbogenendoprothetik nach gescheiterter Versorgung einer Monteggia-like-Fraktur genauer zu evaluieren.

## Fazit für die Praxis

Diese Fallserie zeigt, dass chronische Infektionen und knöcherne Defekte des Processus coronoideus ulnae die häufigsten Ursachen darstellen, die nach gescheiterter Behandlung einer Monteggia-like-Verletzung zur sekundären Implantation einer Ellenbogentotalendoprothese führen. Es handelt sich hierbei um eine „Salvage“-Operation, die insgesamt zu zufriedenstellenden klinischen Kurzzeitergebnissen führt, allerdings mit einer hohen, kurzfristigen Revisionsrate einhergeht und daher in ihrer Indikation streng selektioniert werden muss.
